# Correction: Deagrarianisation and Forest Revegetation in a Biodiversity Hotspot on the Wild Coast, South Africa

**DOI:** 10.1371/journal.pone.0100463

**Published:** 2014-06-11

**Authors:** The PLOS ONE Staff

## Notice of Republication

This article was republished on May 22, 2014, to correct errors in figure legends that occurred during the typesetting process. The publisher apologizes for these errors. Please download this article again to view the correct version. The originally published, uncorrected article and the republished, corrected article are provided here for reference.

## Supporting Information

File S1 Originally published, uncorrected article.
(PDF)

File S2 Republished corrected article.
(PDF)
