# Supplementary material for: Correction: Deagrarianisation and Forest Revegetation in a Biodiversity Hotspot on the Wild Coast, South Africa
Source: PLoS One. 2014 Jun 11;9(6):e100463. doi: 10.1371/journal.pone.0100463 (PMC4053419; doi:10.1371/journal.pone.0100463)
Supplement: File S2 — Republished corrected article. (PDF) [file pone.0100463.s002.pdf]

Deagrarianisation and Forest Revegetation in a Biodiversity Hotspot on the Wild Coast, South Africa

Ross Shackleton, Charlie Shackleton*, Sheona Shackleton, James Gambiza

Department of Environmental Science, Rhodes University, Grahamstown, South Africa

Abstract

Deagraianisation is a worldwide phenomenon with widespread social, ecological and economic effects yet with little consensus on the local or higher level causes. There have been contested views on the causes and consequences of deagrarianisation on South Africa's Wild Coast, which is an international biodiversity hotspot. Using GIS, household interviews and ecological sampling, we compared the perspectives of current and former cultivators as to why some have abandoned farming, whilst also tracking the uses and woody plant cover and composition of fields abandoned at different periods. The GIS analysis showed that field abandonment had been ongoing over several decades, with a decline from 12.5 % field cover in 1961 to 2.7 % in 2009. The area of forests and woodlands almost doubled in the corresponding period. There was a distinct peak in field abandonment during the time of political transition at the national level in the early 1990s. This political change led to a decrease in government support for livestock farming, which in turn resulted in reduced animal draught power at the household and community level, and hence reduced cropping. The study showed it is largely the wealthier households that have remained in arable agriculture and that the poorer households have abandoned farming. The abandoned fields show a distinct trend of increasing woody biomass and species richness with length of time since abandonment, with approximately three woody plant species added per decade. Most local respondents dislike the increases in forest and woodland extent and density because of anxiety about wild animals causing harm to crops and even humans, and the loss of an agricultural identity to livelihoods and the landscape.

Citation: Shackleton R, Shackleton C, Shackleton S, Gambiza J (2013) Deagrarianisation and Forest Revegetation in a Biodiversity Hotspot on the Wild Coast, South Africa. PLoS ONE 8(10): e76939. doi:10.1371/journal.pone.0076939

Editor: Matt Hayward, Bangor University, United Kingdom

Received July 2, 2013; **Accepted** September 5, 2013; **Published** October 14, 2013

Copyright: © 2013 Shackleton et al. This is an open-access article distributed under the terms of the Creative Commons Attribution License, which permits unrestricted use, distribution, and reproduction in any medium, provided the original author and source are credited.

Funding: This work was funded by Rhodes University. The funders had no role in study design, data collection and analysis, decision to publish, or preparation of the manuscript.

Competing interests: The authors have declared that no competing interests exist.

* E-mail: c.shackleton@ru.ac.za

Introduction

Deagrarianisation is a widespread phenomenon that significantly affects local and national food security, rural livelihoods and the environment [1,2]. Globally, abandoned crop land increased from approximately 50 million ha in the 1940s to over 200 million ha by 1990 [3]. Drivers of abandonment of farming as a livelihood include land degradation and declining soil fertility, increasing prices of farming inputs, the migration of rural inhabitants to urban areas and the inability of small-scale farmers to compete with large-scale conglomerates [1,2,4]. Nonetheless, with increasing human populations and westernisation of consumption patterns any decrease in agricultural production and hence food security is usually viewed with concern at both local and national scales [5]. Additionally, field abandonment may be associated with increased wild fires, soil erosion, and the establishment of invasive species [5,6]. However, if native plant communities re-establish on abandoned fields it can help reverse local-scale loss of biodiversity and associated ecosystem goods and services of which agriculture is a primary driver [7].

Deagrarianisation on South Africa's Wild Coast

Agriculture on the South African Wild Coast has declined over the last 70 years. As of the 1940s, maize cultivation declined as a result of several factors, including the pressure of state subsidies and aid to white farmers, price and quality controls, increasing land taxes, and the large-scale recruitment of unskilled labour to work in gold mines [8,9]. Later, the forced relocation of formerly dispersed homesteads into concentrated villages (under the so-called national "Betterment" programme) resulted in increased distances between homesteads and fields for the majority and the resultant increase in small-scale garden cropping [8-10]. Field sizes decreased from an average of 1 327 m^2 in the early 1940s to 650 m^2 in the early 1980s [11], and abandonment rates of agricultural fields ranged from 20 % to 100 % across different villages [11,12]. Currently, agriculture does not meet the food needs of most households

and little produce is sold [13-15]. There has also been a decrease in livestock ownership [11,12]. This may also constrain cropping because of insufficient draught power [16]. More recently, several commentators advocate that the primary driver of the decline is the increase in disposable cash income from migrant labour and state social grants and pensions so that rural people now purchase food in urban markets rather than grow their own [13-15]. Additional reasons for field abandonment along the Wild Coast have been mooted as exhausted soils, unpredictable weather, lack of labour due to children attending school and the migrant labour system to large cities and mines, lack of access to credit and income to buy capital inputs, and problems with livestock and wild animals destroying crops [8,11,12].

The overall decrease in farming has led to the reduction of community ethics and identity related to farming [11]. This is shown in the lack of consensus and kinship of households preventing their livestock from entering a neighbour's field [11]. Nonetheless, although farming usually does not provide for all of a household's food needs, it is still a potentially important source of nutrition and food security and is useful for freeing up cash and acting as a safety net during vulnerable times, especially for the rural poor [15,17,18]. Further advantages can be found in its role of building social capital, maintaining traditions, providing animal feed during winter, as well as to reinforce land ownership and entitlement [12].

Field abandonment and revegetation

After abandonment, old fields may revert to some semblance of natural vegetation, or may be suspended in an intermediate or even degraded state. This depends upon the nature and intensity of the previous farming activities, post-abandonment land uses and disturbances, the quantity and quality of the seed bank, proximity and quality of nearby natural vegetation and the abundance of seed dispersal agents [2,19-21]. Where conditions and management are conducive old fields may revegetate along an identifiable trajectory [22], and is commonly associated with a significant increase in plant biomass [23], although not necessarily an increase in plant species richness [24]. The rate of change depends upon the frequency and intensity of disturbance and the duration of the pioneer stage. In many places in South Africa *Acacia karroo* is a dominant pioneer tree species, and woody plant community composition has not moved past the *A. karroo* stage even after 50 years [20,25,26]. Berliner [27] suggests that the same applies on the Wild Coast, where *A. karroo* establishes in abandoned fields, but few other woody species follow. However, others see it is a traditional pioneer species which after three or four decades provides conducive conditions for other woody species to establish [28,29]. Whether this ultimately moves to a plant community structure and composition similar to relatively intact forests of the region remains unclear.

Forests on the Wild Coast

Within South Africa the forest biome is the smallest of the seven biomes, accounting for less than 0.2 % of the terrestrial area [30]. Forests along the Wild Coast form part of the Maputuland-Pondoland-Albany biodiversity hotspot. There are approximately 50 000 ha of indigenous forest along the Wild Coast, largely made up of numerous fragments smaller than 100 ha within a grassland matrix [27], maintaining high faunal richness [31]. The size and distribution of the forest patches is dynamic, being influenced by soil factors and fire regimes as well as fluctuating intensity of human uses over millennia [10,32,33].

The Wild Coast forests provide multiple benefits to local people. Direct benefits include building materials, fuelwood, wood for other purposes, medicinal plants, wild foods, grazing, recreation, bushmeat, crafts and areas of cultural importance [27,30,34-36]. Indirect benefits include aspects such as water provision, carbon sequestration, climate regulation and cultural sites [37]. Despite these benefits, some commentators argue that forests are being lost at an alarming rate due to increased human population and the associated land use pressure, a reduction in the authority of traditional leaders who previously played a key role in managing forests and regulating fires, mining and the impacts of invasive vegetation [27,30,34,38]. However, counterviews suggest that the increase in the number of state social grants, fewer livestock, and greater access to electricity have resulted in declining direct use of the forests and consequent natural reforestation in some parts of the hotspot (10, 39-40]. Increased atmospheric carbon dioxide due to climate change may also be playing a role in promoting woody species presence and growth [41,42].

From the above a number of contested views are apparent relating to the causes and consequences of deagrarianisation on the Wild Coast, especially (i) what are the primary drivers of abandonment of farming livelihoods, (ii) level of use and loss of forests and (iii) whether abandoned croplands remain dominated by *Acacia karroo* or revert to forest. Some of these differences may well lie in the limited integration of social and ecological perspectives [10]. Most studies have focused only on drivers and the impacts of deagrarianisation for humans with limited consideration of the environmental effects [11,12], and GIS studies have not ground-truthed the species composition of reforesting areas [10,39]. Consequently, this study sought to understand the interplay of socio-economic drivers and ecological consequences of deagrarianisation on the Wild Coast. Key questions related to (i) causes of deagrarianisation and forest change, (ii) perceptions of such changes, (iii) whether succession after field abandonment leads to an increase in species richness over time, and (iv) local perceptions, practices, preferences and use of different landscapes.

Study Site

The Willowvale area (32.26° S; 28.50° E) is located in the Eastern Cape province of South Africa along what is known as the Wild Coast. The area is dominated by rolling hills and valleys, with an altitude ranging from sea level to 450 m [43]. The rainfall is approximately 800–1 000 mm per annum, concentrated in summer (October to April), although some winter rain is common. Mean temperatures range from highs of 27 °C in summer to 3 °C lows in winter.

The geology in the area is of the Karoo Supergroup, consisting of mudstones of the Adelaide Subgroup and shales, mudstones and sandstones of Beaufort and Ecca Group and tillites of the Dwyka group [43]. Moderate to deep apedal Glenrosa and Mispah soil types dominate making up sandy and clay loams [43]. The natural vegetation is a mosaic of forest, thornveld, dune thicket and grassland patches [43]. The area is a meeting point of four of the seven biomes found in South Africa [44]. It falls within the Maputaland-Pondoland-Albany biodiversity hotspot, characterised by high levels of endemism and threatened species [44]. The forests are the most species rich non-tropical forests in the world [44]. With high endemism and local land use pressures it is regarded as a priority corridor for biodiversity conservation [44].

According to the local municipality approximately 91 % of households live below the poverty line [45]. Illiteracy is about 56 %, and unemployment approximately 79 % [45]. Population density is approximately 53 persons per km^2. Land tenure is communal, controlled by chiefs and tribal authorities. The landscape has patches of grazing land, subsistence farming and dispersed homesteads [44]. There is high reliance on state welfare grants and remittances from migrant labour for cash income [12]. The livelihoods of these villagers are diversified with a combination of cash incomes, state grants, livestock, subsistence farming, petty-trading, micro-enterprises, and collection of non-timber forest products (NTFPs) [8,35].

Methods

Several methods were employed to address the key questions. This study was approved by the Dept of Environmental Science Ethics committee, and the broader study of which it was part was approved by the Rhodes University Ethics committee. Permission to work in the area was granted by the local Traditional Authority. Given high levels of illiteracy amongst the interviewees, verbal, rather than written, informed consent was sought and noted. Where verbal consent was not granted (one instance) no interview was conducted.

Assessing changes in land cover/use

To assess broad trends in landscape use and cover, 1961 aerial photos were compared to 2009 photos by means of randomly selected sample plots (50 m x 50 m). Eight hundred and forty-four plots were sampled on the 1961 photos and 949 on the 2009 photos, representing approximately 5 % of the study land area. Each plot was categorised by its dominant land use or cover, i.e. abandoned garden, abandoned field, current field, current garden, forest, woodland, and grassland. Woodlands were identified as areas dominated by woody vegetation but with a markedly discontinuous canopy, whilst forests were dominated by woody vegetation with a canopy cover of greater than 90 %.

Assessing vegetation succession in abandoned fields

Woody plant density and composition were assessed in fields abandoned between 1940 and 2000. Old fields were selected and aged using aerial photographs and triangulated with information provided by local inhabitants. Forty-five (10 x 20 m) plots were sampled in abandoned fields; three plots in fields abandoned in the 1940s, seven in the 1950s, five in the 1960s, 15 in the 1970s, nine in the 1980s and six in the 1990s. A further ten were sampled in uncleared forest as a reference. In each plot the GPS coordinates, slope (Abney level), altitude (GPS), aspect (compass), slope position (six point scale) and the distance to the nearest forest were recorded. The total woody cover was estimated visually. The species and basal diameter at 30 cm above ground level for all stems thicker than 1 cm were measured and woody species smaller than that were counted.

In each 200 m^2 plot four 1 x 1 m quadrats were used to visually estimate total percentage cover of herbaceous plants, grass, bare ground and organic litter. The 1 m^2 quadrats were placed in the centre of each quarter of the 200 m^2 plots. A penetrometer reading in each of the four 1 m^2 quadrats was taken to assess soil compaction. Soil samples from each of the four 1 m^2 quadrats were taken to assess soil organic carbon (%), P (mg/l), exchangeable acidity (cmol/l), total cations (cmol/l), acid saturation (KCl), pH (KCl), Zn (mg/l), K (mg/l), Ca (mg/l) and Mg (mg/l). The top 1 cm was removed, and a sample to the depth of 10 cm was taken. The four soil samples within each 200 m^2 plot were pooled.

Local perceptions regarding farming, field abandonment and forest regrowth

In May/June 2013 a household questionnaire was administered to current and former cultivators (people who had or have fields, as opposed to small vegetable gardens adjacent to the homestead) to understand people's beliefs about changes in land use practices, the causes thereof and their perceptions of what happens to abandoned fields. The questionnaire also assessed local ecological knowledge regarding woody species change in abandoned fields. Fifty interviews were done with people who no longer cultivate fields and 31 with households who still cultivate. A snowball sampling approach was adopted to identify households in each category. Abandonment of farming livelihoods and croplands was strongly evident in the study area, with only a small proportion of households still cultivating fields, necessitating that the original study area had to be more than doubled from 14 to 38 villages to get an a sample of at least 30 field cultivators. The oldest person in the household was interviewed as there were questions relating to changes in practices and fields over time. A household was loosely deemed to be all people who ate and slept in the homestead most of the time or if away made regular remittances to the household.

Data analyses

Independent t-tests and chi-squared tests were used to compare the differences between households that farm and those who have ceased farming. Regression analysis was used to assess changes in woody plant species richness, diversity (Shannon-Wiener), basal diameter and soil characteristics relative to age of abandoned fields. In these regressions the age of uncleared forests was set as 100 years (changing this age to 80, 120 or 150 years had negligible

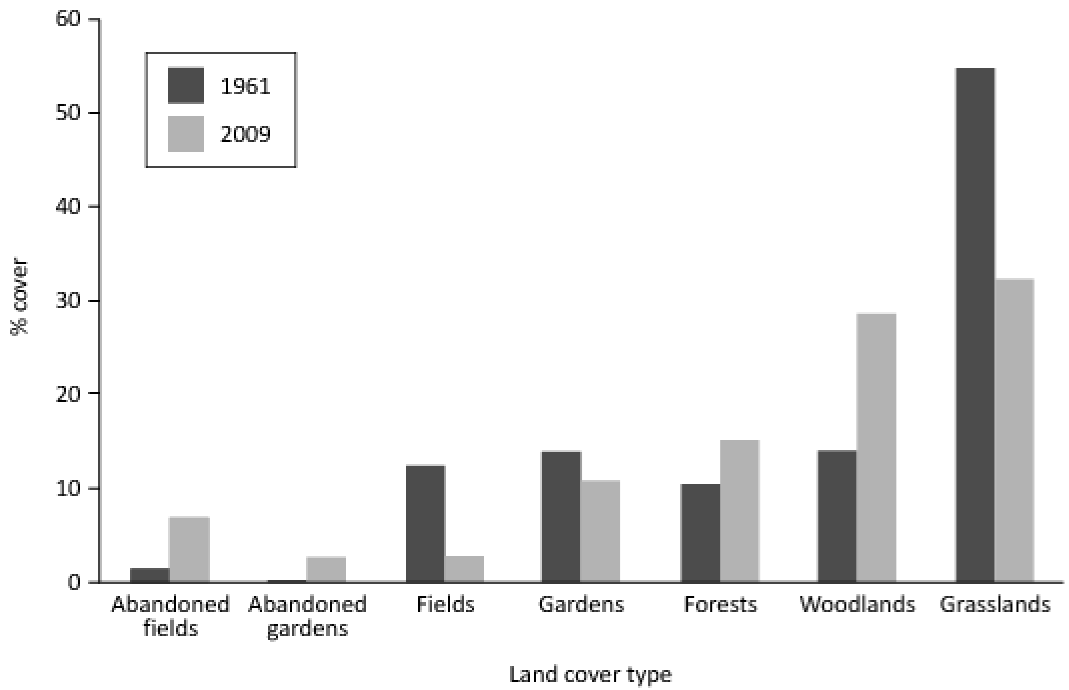

Figure 1. Comparison of land cover between 1961 and 2009 (n = 844 and 949, respectively).

doi: 10.1371/journal.pone.0076939.g001

effects on the resultant regressions). Detrended Correspondence analysis (DCA) was used to analyse total woody plant species composition relative to age of the abandoned fields. The Rand-US dollar exchange rate at the time of the study was approximately US $ 1 = R 8.15.

Results

Land cover/use change

The GIS analysis showed that the landscape had changed substantially over the past 50 years (Figure 1). Grassland reduced by 22.5 %, whereas woodland increased from 13.9 % in 1961 to 28.8 % in 2009 and forests increased by 5 % (χ^2 = 21.3, p = 0.0002). There was a large reduction in field cover from 12.5 % in 1961 to 2.7 % in 2009 and an increase in abandoned field cover from 1.5 % in 1961 to 6.9 % in 2009. There was also increasing abandonment of home gardens but not to the same extent as fields. This links well to the interview responses where people commonly mentioned that there had been a loss of fields and increased woody cover in the area. It also illustrates that forests are encroaching into grasslands and not only abandoned fields.

Revegetation of abandoned fields

Most of the vegetation attributes measured in the field plots demonstrated clear trends with time since field abandonment (Table 1). The number of woody species ($r = 0.8$, $p < 0.0005$) and basal area ($r = 0.8$, $p < 0.0005$) increased with abandonment time of fields. Woody cover and tree density also increased, whilst grass cover decreased significantly as old field plots became more wooded. The amount of bare ground increased with age of abandonment, which is most likely linked to less grass. Litter cover also increased with age of field abandonment, being about six times higher in fields abandoned in 1945 compared to those abandoned in 1995. There was an association of lower slope angles and field abandonment dates, possibly reflecting that it is easier to farm on gentler slopes and so those were the last fields to be abandoned. Other variables such as distance to the nearest forest, aspect, slope position and percentage herbaceous plant cover showed no trends over time.

In the uncleared forests the seven most common woody species contributed less than half (43 %) of total tree density, whereas in fields abandoned in the 1990s the most common species (*A. karroo*) contributed 83.9 % of tree density (Table 2). There was a steady decrease in relative contribution of *A. karroo* with age since abandonment. Fields abandoned in the 1940s, 1950s and 1960s had many dead *A. karroo* stems.

Woody plant species diversity increased from 0.8 in fields abandoned in 1990s to 3.13 in fields abandoned in the 1950s, approaching that of uncleared forests, which had a Shannon-Wiener diversity index of 3.58 (Figure 2). The oldest abandoned sites were not as diverse as the forests, but close. The mean number of woody species in the forest plots was 26.6 ± 5.6 compared to 20.0 ± 6.1 in the old field sites abandoned the longest (Table 1). The regression of species richness against time indicated that approximately three woody species were added per decade after abandonment (Figure 2). Alien invasive species made up 17.8 % of the top seven most common species in abandoned fields and forests (Table 2).

Table 1. Physical and vegetation attributes (mean ± SE) of uncleared forests and fields with different lengths of abandonment (N = 55).

Variable		Decade abandoned:						Statistics:	
	Forest	1940s	1950s	1960s	1970s	1980s	1990s	r	P
Distance to nearest forest (m)	0	6±41	108±108	158±96	183±212	150±40	300±125	0.3	0.37
Altitude (m.asl)	236±165	199±41	271±117	239±131	230±134	206±90	263±130	0.0	0.94
Aspect (°)	180±115	132±110	78±91	125±121	168±129	161±119	191±96	0.0	0.93
Slope position	3±0	3±1	3±1	2±1	3±1	4±2	3±2	0.3	0.85
Slope angle (°)	21±8	22±6	17±7	19±8	17±8	10±7	7±6	0.5	0.002
Tree Density (no./200 m^2 plot)	198±38	227±43	355±245	143±48	154±116	73±36	45±37	0.4	0.008
No. woody species (no./200 m^2 plot)	26±5	20±6	22±6	18±7	13±7	7±4	4±3	0.8	<0.005
Basal area (m^2/ha)	52±23	19±6	18±10	16±12	14±10	4±3	2±3	0.8	<0.005
Woody cover (%)	98±3	83±5	81±42	54±22	45±26	17±15	10±12	0.8	<0.0005
Grass cover (%)	1±1	20±6	27±19	60±28	59±28	69±24	81±9	0.8	<0.005
Herb cover (%)	24±8	10±6	22±9	21±17	13±8	20±20	18±19	0.2	0.21
Litter cover (%)	50±14	44±11	28±12	19±16	15±14	4±4	6±5	0.8	<0.005
Bare ground cover (%)	27±17	13±13	21±15	13±12	12±10	11±12	8±5	0.5	0.004

doi: 10.1371/journal.pone.0076939.t001

Table 2. The relative density (%) of the most common woody species in forests and across fields with different abandonment dates (N=55).

Species		Decade abandoned:					
	Forest	1940s	1950s	1960s	1970s	1980s	1990s
Acacia karroo	0	2.7	5.6	18.1	24.3	54.4	83.9
Acalypha glabrata	6.5	0	0	0	0	0	0
Calpurnia aurea	3.2	0	3.4	0	0.04	0	0
Cestrum laevigatum *	2.2	0	0	1.23	0.2	1.3	1.1
Coddia rudis	1.1	11.1	16.3	12.7	8.9	9.3	1.5
Diospyros dichrophylla	0.2	2.1	2.1	2.1	4.4	2.6	4.1
Diospyros lycioides	3.9	12.3	11.9	8.4	6.2	4.3	0
Grewia occidentalis	2.9	7.5	4.9	6.3	2.9	2.2	0.3
Justicia campylostemon	5.7	0	0	0	0	0	0
Lantana camara *	2.9	16.7	12.6	13.0	25.9	15.9	3.0
Maesa alnifolia	1.3	1.8	1.1	4.3	2.2	5.6	1.1
Millettia grandis	7.7	0.7	0.9	0	0.3	0	0
Plectranthus ecklonii	5.5	0	0	0	0	0	0
Scutia myrtina	0	3.7	0.5	0.4	0.3	0	0
Solanum chrysotrichum *	4.6	0	0	0	0.1	1.4	0
Tecomara capensis	0	0	3.2	0.4	3.1	0	0
Tricalysia lanceolata	8.6	6.4	3.7	0.6	2.7	0	0
Trimeria grandifolia	0.9	10.4	2.7	3.9	2.3	0.2	0
Zanthoxylum capense	4.4	5.1	3.2	5.7	2.2	2.2	1.5

doi: 10.1371/journal.pone.0076939.t002

The DCA revealed five distinct groupings arranged along the first axis, corresponding to abandoned fields of different ages and one cluster for uncleared forests (Figure 3).

Soil carbon increased significantly with increasing age of the abandoned fields (Table 3), as did total cations and calcium. Soil compactness decreased. All the other soil attributes showed no trends in relation to age of abandoned fields.

Characteristics of current and past cultivators

Just under half (46 %) of the respondents were male and the average age of respondents was 59 ± 14.9 years. Poverty was extensive in the area resulting from limited employment opportunities and low education levels (and average of 4.4 ± 4.3 years of schooling). Only 19.8 % of households had electricity. Cultivating households were larger (7.4 ± 4.0 people per household) than past-cultivators (5.6 ± 2.9) ($t = 2.3$, d.f = 79, $p = 0.03$). The majority of households' cash income came

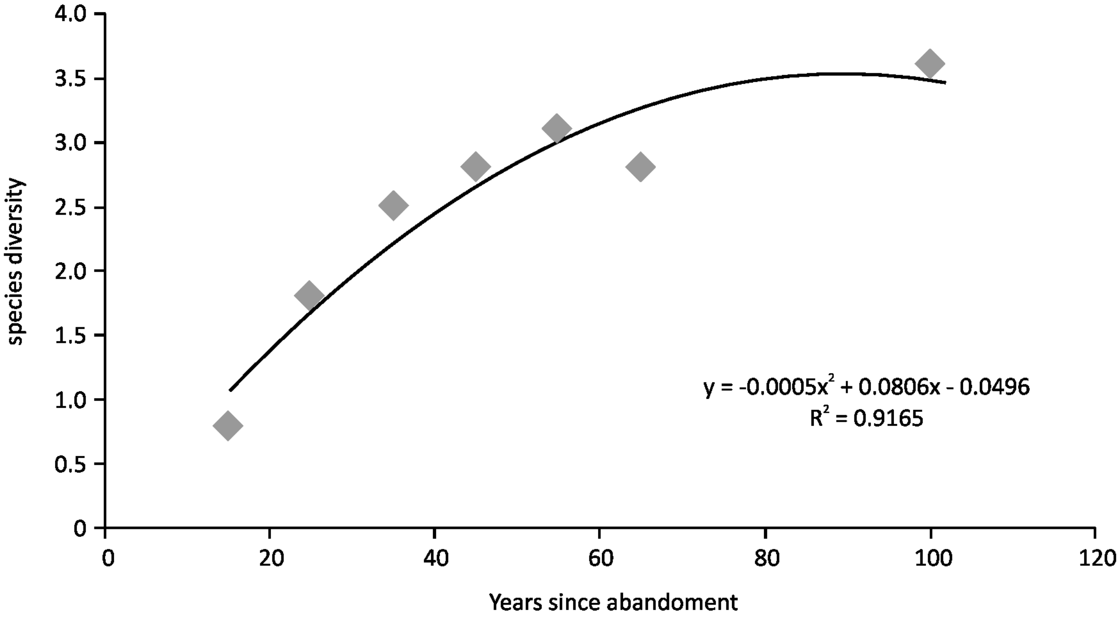

Figure 2. Shannon-Wiener species diversity change with time of field abandonment (forests set at 100 years) (n = 55).
doi: 10.1371/journal.pone.0076939.g002

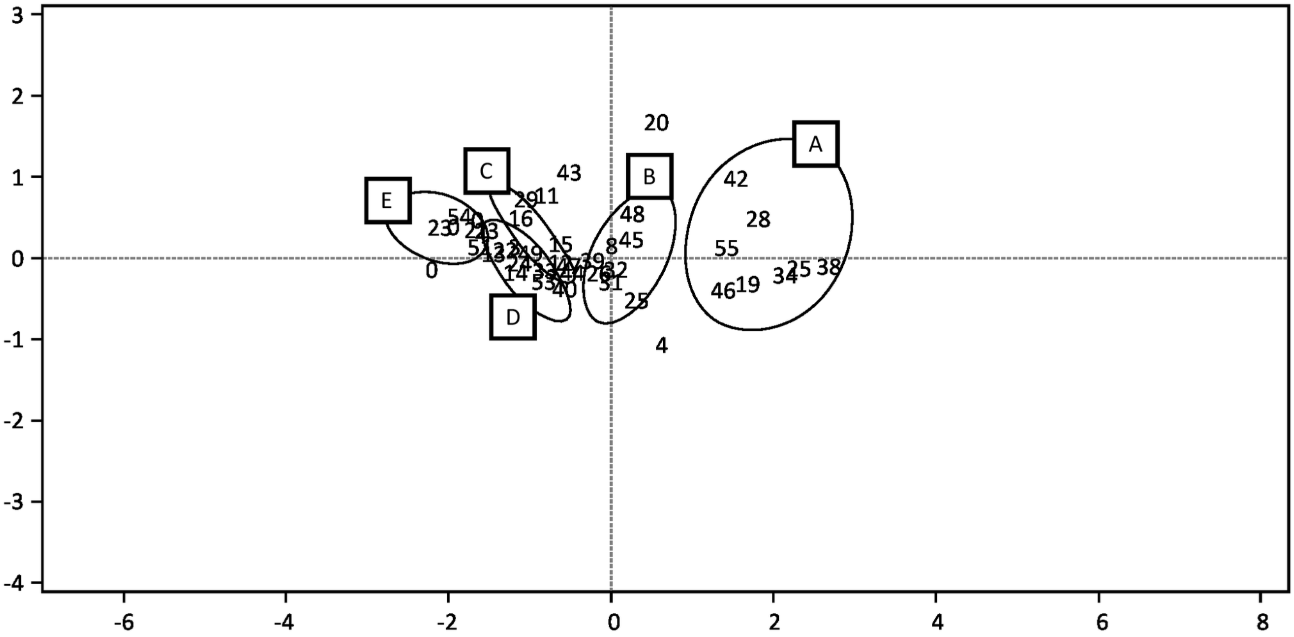

Figure 3. Detrended Correspondence Analysis (DCA) ordination diagram of plots for species composition in fields with different abandonment decades and intact forests. (A = forests; B = 1940s and 1950s plots; C = 1960s; D = 1970s and 1980s; E = 1990s). (n = 55)
doi: 10.1371/journal.pone.0076939.g003

from state welfare grants and pensions. Current cultivators averaged 1.9 ± 0.7 grants and 0.8 ± 1.7 state pensions per household, which was similar to past cultivators who received 1.4 ± 1.5 grants and 1.1 ± 1.2 state pensions per household. However, farming households had significantly higher employment rates with an average of 0.5 ± 0.6 jobs per household, compared to former cultivators which had only 0.2 ± 0.4 jobs (t = 2.1, d.f = 79, p = 0.04). Cultivating households

Table 3. Soil characteristics (mean ± SE) with age of abandoned fields and uncleared forests (N = 55).

Variable		Decade abandoned:						Statistics:	
	Forest	1940s	1950s	1960s	1970s	1980s	1990s	r	p
Organic carbon (%)	4.0±0.6	4.3±0.7	3.4±0.9	2.9±0.9	2.8±1.0	1.8±0.7	1.6±0.6	0.7	<0.001
pH (KCl)	4.8±0.7	5.4±0.2	4.9 ±0.5	4.7 ±0.5	4.8±0.4	4.6±0.2	4.6 ±0.6	0.2	0.214
Exch acidity (cmol/l)	12.5±34.2	0.2±0.0	0.2±0.1	0.5±0.5	0.7±0.8	0.2 ±0.1	0.3 ±0.1	0.3	0.058
Acid saturation (KCl)	9.5±9.7	2.0±0.0	2.1±1.0	8.0±9.1	7.3±8.0	3.4±1.9	5.2±2.1	0.2	0.223
Total cations (cmol/l)	7.7±1.4	9.7±1.6	7.6±1.3	7.3±2.3	6.9±2.1	5.7±2.3	5.6±1.7	0.3	0.014
K (mg/l)	114±53.1	164±36.2	101±31.4	130 ±21.0	103±36.5	69±40.9	133±119.7	0.1	0.451
Ca (mg/l)	858±403.7	1138±337.6	878±236.6	721±543.4	646±307.7	593±296.9	549±344.6	0.3	0.027
Mg (mg/l)	302±72.4	415±13.7	337±85.3	356±39.5	349±66.5	317±125.1	277±34.2	0.0	0.952
P (mg/l)	25.4±8.8	15.3±2.4	15.0±6.3	11.0±12.2	19.0±11.3	13.8±9.7	21.0±6.3	0.2	0.082
Zn (mg/l)	7.6±6.9	6.2±5.2	5.1±7.2	14.7±8.1	8.0±12.2	4.4±5.4	10.2±12.3	0.0	0.915
Penetrometer	2.5±0.8	3.0±1.3	4.0±0.7	4.2±0.3	4.1±0.5	4.4±0.2	4.0±1.2	0.7	<0.001

doi: 10.1371/journal.pone.0076939.t003

received an average of 0.4 ± 0.7 remittances per household, whereas past cultivating households received 0.2 ± 0.4 remittances.

Cultivators had significantly (d.f = 79, p<0.0005) more livestock per household (11.5 ± 9.9 cattle, 14.2 ± 14.9 goats, 9.3 ± 14.9 sheep) than past cultivators (2.2 ± 3.2 cattle per household, 1.7 ± 1.3 goats and 0.1 ± 0.42 sheep). This shows that current field cultivators also seem to be small-scale livestock owners. Current cultivators had more vehicles than past cultivators (0.4 ± 0.7 and 0.02 ± 0.1, respectively (t = 3.5, d.f = 79, p = 0.0006)). Overall, these results indicate that cultivating households were wealthier than former cultivators, as they had greater employment rates, more remittances, assets and livestock.

Farming practices

Many households that had stopped farming did so approximately 18 ± 12 years ago. Current cultivators had cleared their fields more recently (30.2 ± 16.8 years ago) than past cultivators (37.7 ± 16.7 years ago) (t = 2.1, d.f = 79, p = 0.037). On average, 64.5 % of cultivating households said they had cleared primary forest to create their fields, whereas fewer (55.5 %) past cultivators had cleared forest (χ^2 = 4.4 p = 0.03). All past and current cultivators planted maize, 62 % planted pumpkins and 42.5 % planted beans. Less commonly mentioned crops included sweet potatoes, butternut, cabbage and beetroot. Of the households that had stopped cultivating and had not done so for many years, 70 % said other villagers must ask permission to graze livestock or collect NTFPs in their old fields and only 1.2 % stated that they lose the ownership or usufruct of their fields if they do not cultivate them. This suggests a strong sense of entitlement for this land even if it is not cultivated.

Of those households still farming 45 % did so solely to supply food for household use, whilst 55 % grew for own consumption and sale combined. On average, 26.8 ± 21.7 bags (50 kg bags with the corn on the cob) of maize were produced per household per year. The average income from selling extra produce was R 1 450 ± 1 440 per household per year. The average selling price per bag was R 150.

Drivers of field abandonment

The primary reason noted by both groups for reduced agriculture was that ploughing was difficult because residents no longer invest in cattle or their cattle have died (Table 4). Interestingly, some past cultivators (22 %) stated that raiding of crops by livestock was an important factor in driving some households to abandon farming, whereas none of the current cultivators identified this as a cause of deagrarianisation. Similarly, one in ten current cultivators mentioned that many people ceased farming because farm work is too hard and they are ‘lazy’, whereas none of the past cultivators mentioned this. Responses from the prompted list echoed those of the free response list, including not enough equipment and problems with pests and livestock raiding crops. Almost two-thirds (64 %) of former cultivators said that farming is not worth the effort, which may link to the statement regarding ‘laziness’ made by current cultivators.

Approximately 60 % of past cultivators indicated they would be interested in farming again within the next five years. Key reasons were that they wanted to make money and improve food security. However, 56 % of those saying they would be interested have not farmed for 15 years or more. Most of these respondents were waiting for government to provide communal tractors, fertilizer and seed. The rest (40 %) said they would never farm again, mainly because they felt it was not worth it, or they were too old or infirm.

Only one-quarter (25.8 %) of current cultivators believed that the youth would become involved in farming in the future, which is significantly higher than the 6 % of former cultivators feeling the same (χ^2 13.78, p = 0.0002). Forty-five per cent responded that the youth are too lazy to farm and 11 % stated that the youth were moving away to cities. Other reasons were that the youth don’t know how to farm, they were not interested in farming, that they no longer like maize as they prefer to eat rice, it would be too much effort to clear trees from abandoned fields and the soil is poor. Those that responded positively

Table 4. Reasons why people have stopped cultivating (% of responses).

Reason	Current cultivators (n=31)	Past cultivators (n=50):	
		Free response	Prompted response
No cattle	39	54	N/A
Wild animals eat crops	16	12	80
Livestock eat crops	0.0	22	80
Lazy	11	0	N/A
Not enough equipment	N/A	N/A	56
Inputs too expensive	N/A	N/A	56
Not enough labour	N/A	N/A	38
Not worth it/ disinterested	N/A	N/A	64
Have enough food/income	N/A	N/A	14
Unpredictable weather	N/A	N/A	20
Exhausted soils	N/A	N/A	24

doi: 10.1371/journal.pone.0076939.t004

Table 5. The proportion (%) of current (n=31) and past cultivators (n=50) that collect NTFPs from three different land use types (N = 81).

Resource	Forests:		Used and abandoned fields:		Grasslands and homesteads:	
	Current cultivators	Past cultivators	Current cultivators	Past cultivators	Current cultivators	Past cultivators
Fuelwood	97	92	64	64	0	0
Wood for other purposes	81	74	0	6	0	0
Wild fruits	84	74	0	2	0	4
Wild vegetables	0	4	51	6	87	94
Bush meat	45	20	0	0	0	0
Thatch grass	10	6	32	46	58	68
Grass/stick brooms	26	42	13	4	51	58
Medicinal plants	77	76	23	10	16	4

doi: 10.1371/journal.pone.0076939.t005

discussed that the youth would farm to make money and improve food security for their households.

Use of old fields and forests

All cultivators and past cultivators used at least two NTFPs. On average, 20.4 % of cultivating households and 15.3 % of past cultivating households used current and abandoned fields as their primary site for NTFP collection. In comparison, 47.7 % of current and 44.0 % of past cultivators used forests as their primary source of NTFPs. Corresponding figures for around homesteads and adjacent open grassland areas were 23.7 % and 25.3 %, respectively. The proportion of households collecting NTFPs and the variety collected by current cultivators in current and abandoned fields was higher than past cultivators (χ^2 = 60.85, p < 0.0005), the same can be said for forests (χ^2 = 13.1, p = 0.04), as well as homesteads and grasslands (χ^2 = 17.3, p = 0.002).

Respondents mentioned that the NTFPs are less commonly used nowadays compared to the past (Table 5). This was because people have cash incomes (largely from state grants) to buy fuel for cooking (paraffin or gas) and meat and vegetables. They also have better access to clinics when ill and so make less use of medicinal plants from the wild. Whilst most households still used NTFPs, the frequency of use was a lot less, which they perceive to be a factor contributing to forest and woodland change in the area.

Perceptions of forest and woodland change

All current cultivators and 94 % of past cultivators perceived the forest and woodlands near their homestead to be changing, with 96.8 % of cultivators and 88 % of past cultivators saying that they were increasing. Current cultivators said that the forests and woodlands started changing 19 ± 10.6 years ago, whereas past cultivators viewed this change as starting slightly further back, approximately 22 ± 13.9 years ago. These estimates coincide with the mean date of cessation of field cultivation (18 ± 12) years ago.

The perceived key drivers of the forest and woodland increase were a reduction in farming and reduced collection of NTFPs (Table 6), whilst a small proportion (10 %) attributed it to increased rainfall. Cultivators also mentioned the migration of people to cities, which led to decreased use of forests and woodlands, along with a concomitant rapid increase in cover of invasive species. There were significantly different responses between cultivators and non-cultivators for reasons driving forest and woodland increase (χ^2 = 36.6, p < 0.001).

Table 6. Reasons provided by current and past cultivators regarding why forests are increasing (N=74).

Reasons	Proportion of responses (10 % and above):	
	Current Cultivators	Past Cultivators
	(n=30)	(n=44)
Less farming	40	42
Using less NTFPs	30	44
More rain	11	10
Invasive species	10	0
People moving to cities	10	2

doi: 10.1371/journal.pone.0076939.t006

Table 7. Widely mentioned woody species that establish first into abandoned fields as mentioned by current and past cultivators (N=81) (* = invasive alien species).

Species	Current cultivators (%) (n=31)	Past cultivators (%) (n=50)
Acacia karroo	100	88
Lantana camara *	36	20
Diospyros lycioides	19	14
Cestrum laevigatum *	13	12
Millettia grandis	0	22
Acacia ataxacantha	19	0

doi: 10.1371/journal.pone.0076939.t007

The majority (83 %) of both groups felt that the increase was undesirable; the most common reason being because it increased the presence of wild animals that are dangerous to humans and eat crops. Commonly cited dangerous animals were snakes and jackals, whilst bush pigs, jackals, baboons, monkeys and rats were seen to raid chickens and crops. Other negative perspectives related to forest and woodland increase were that criminals hide there (36.4 % for past cultivators and 24.1 % for current cultivators) and that it represents loss of faming in the area (13.8 % current cultivators and 4.6 % past cultivators). Only a few viewed the increase in a positive light, stating that it was beneficial because NTFPs were becoming more abundant (10.3 % for current cultivators and 13.6 % past cultivators). Fifty-two per cent of past cultivators mentioned that their abandoned fields have become forest or woodland, 36 % said that they were now grasslands, and 8 % said their field had become homesteads. Cultivators responded that it takes 0.9 ± 1.6 years for trees less than 10 cm tall to establish in abandoned fields, whereas past cultivators stated it takes 2.1 ± 0.9 years. Both cultivators and past cultivators mentioned it takes 7-8 years for a tree of approximately 1.5 m tall to grow into abandoned fields. Most respondents said that the first and most common woody species to grow into abandoned fields was *A. karroo* (Table 7).

Discussion

Drivers of deagrarianisation

The GIS and household interview results affirm that deagrarianisation has been going on over several decades [10,11,14], although with a noticeable peak approximately 18 ± 12 years ago. This corresponds closely with the election of the first democratic government in South Africa in 1994. With the political transition came accelerated weakening of local traditional governance structures, improvements in state social welfare grants and pensions as well as expectations of development and employment which may have led many to stop farming. The abolishment of racially discriminatory laws segregating living areas also allowed people to move freely. This period also saw the restructuring in agricultural extension services and support from government [46]. Thus, these changes in the macro political landscape altered social paradigms leading to landscape change at the local level.

Loss of cattle was the most common reason mentioned for the abandonment of farming because people then had no means to plough. This led to increases in the extent of woodlands and forests because of reduced agriculture. The reduction in cattle numbers will have also resulted in reduced browsing and trampling which may have increased recruitment of small woody plants. Most people mentioned that most or all of their cattle had died through drought or disease or had been sold but not replaced as people moved away from traditional investment in livestock to monetary savings. This also may be linked to the collapse of agricultural extension services and the reduction of government sponsored veterinary and dipping services, as government priorities shifted to urban development after the political transition [14]. Andrew and Fox [11] and De Klerk [10] show that livestock numbers have decreased but do not attribute this as a reason for a reduction in field cultivation. Lack of capital equipment, problems of crop raiding by livestock and wild animals, expensive inputs and people no longer being interested were also common reasons for abandonment of arable farming. Hebink and Lent [14] discuss the increase in government social grants as being the key driver of field abandonment in the Eastern Cape; however this was not mentioned by respondents in our study. Benayas et al. [47] show that globally, field abandonment is more commonly driven by socio-economic factors than ecological ones, mirrored by this study. Small-scale agriculture is often viewed as being for the poorest of the poor. However, this study shows it is the wealthier households, with higher cash incomes from non-farm sources, which have continued cropping and that the poorer households have abandoned fields. This suggests that economic factors, such as lack of capital and lack of funds to pay for inputs may be core reasons. Wealthier households may also have better access to labour (hired or own), skills and nutrition, which are useful for sustaining arable agriculture.

The respondents saw field abandonment as a long-term trend and that the youth are not likely to partake in arable farming in the future, and at least 40 % of households would never start agriculture again. This is due to a lack of interest and to migration to urban areas [15,48]. Nonetheless, more than half of the respondents who had abandoned their fields

stated that they would be interested in starting again, even though the majority had not farmed for 15 years or more. Key to starting again would be government support such as tractors, seed and fertiliser. Some respondents stated that there are communal tractors available, but that they are controlled by local elites who restrict their availability for use by the wider community. Additionally, maintenance is poor so that the government tractors frequently fall into disrepair.

Land cover change

Over 90 % of respondents reported that forests and woodlands are expanding, which is considerably more than the 40 % reported by Chalmers and Fabricius [39] at a site to the north of Willowvale. In our study 37 % of respondents viewed a reduction in cropping to be the main reason for reforestation as opposed to 75 % reported by [39]. De Klerk [10] also reported the reduction in agriculture to be the key driver of reforestation on the Wild Coast. Households viewed forest and woodland expansion to have accelerated approximately 20 years ago, however, De Klerk [10] showed it to be a longer term trend with woody cover increasing from 56 % in 1961 to 82 % in 2001 in Nqabara. Chalmers and Fabricius [39] found wooded areas to have increased by 49 % between 1974 and 2001 around several villages on the Wild Coast. These two studies show that woody expansion started earlier than perceived by respondents in our study, and mirrors woody biomass increments in other parts of the country under varying tenure and management regimes [49]. Other drivers of forest and woodland increase discussed by locals were reduced forest and woodland use, increased rain in the area, alien invasive species and change in land use practices due to human migration to cities. Chalmers and Fabricius [39] reported that locals mentioned reduced grazing and changes in fire regimes as factors increasing forest growth, neither of which were mentioned in this study. Respondents discussed that not only are forests and woodlands expanding, but that they are also becoming more dense [39].

Most respondents (83 %) viewed the forest and woodland expansion in a negative light, largely because it was associated with increasing populations of wild animals that are dangerous or attack chickens and crops. Other negative perceptions of the expansion included that it represents a loss of agriculture in the area whereas respondents prefer landscapes dominated by productive fields, and that the wooded areas provide hiding areas for criminals. Similar findings were reported from several European countries [50] where the majority of respondents viewed forest increase as undesirable as it was against the wishes of locals, created a sense of isolation between households, represented a loss and threat to farming and decreased the beauty of the area. In Willowvale, a minority of respondents welcomed the forest and woodland increase because it was beautiful and that it increased the availability of NTFPs. Similarly, Elands et al. [50] found some people in Europe appreciated reforestation because it provided income for locals, it increased cultural values as well as improved ecosystem services and improved the beauty in the area.

All respondent households used at least two NTFPs, most commonly from the forests, followed by gardens and grasslands and lastly in fields and old fields. The most commonly collected NTFPs were fuelwood, wood for other purposes (e.g. fencing), wild fruits, wild vegetables, thatch grass and medicinal plants, mirroring results from other studies in the region [35]. Generally, the cultivating households, who were wealthier, used more NTFPs than poorer, former cultivators. In rural areas wealthier households use as many if not more NTFPs than poorer households, however usually in lower quantities and they are often bought rather than collected by household members [51].

Changes in vegetation structure and composition

Other authors [10,39] have showed that the area of woody cover had increased in several areas along the Wild Coast. However, this was done on a broad scale using GIS with no ground-truthing. This led to claims that it was not reforestation, but just *A. karroo* encroachment due to altered fire regimes [26,27]. Our study shows that species richness, diversity, basal area and woody cover increased in old fields with time, on a clear trajectory towards that of natural forests. Forest climax species become common after 45 years although the mean basal area is still less than half that of uncleared forests even 60 to 75 years after abandonment. Species diversity is high in forests and in fields abandoned over 50 years ago, but is low in recently abandoned fields. Approximately three additional woody species established for every decade of abandonment. Reforestation rates vary in relation to time since abandonment as well as local environmental conditions and post-farming land uses. For example, in North America and Central Europe the vegetation structure and species richness approach those of uncleared forests after 20 to 30 years [3].

The results show that concern that the reforestation after field abandonment is just *A. karroo* encroachment is unfounded. *Acacia karroo* is playing a typical pioneer species role, being the first to establish and dominate old fields, declining thereafter. After 40 to 50 years individuals die [29], such that it is only a minor constituent of the woody plant assemblage.

Several alien invasive species were present in revegetating fields (and some uncleared forests), the most common one being *Lantana camara*. It is possible that these invasive species may slow down the revegetation processes by outcompeting native species. Tognetti et al. [52] found that in grasslands in Argentina the presence of invasive species negatively impacted the natural successional process. There is still a need to better understand the role of invasive species on natural vegetation succession [53].

Conclusion

This study has shown that there are linkages between the political, social and ecological factors which interact to drive particular land uses in social-ecological systems and hence landscape appearance on the southern Wild Coast. Deagrarianisation was widespread in the area as a consequence of multiple drivers. A key one appears to be the reduction in cattle numbers which used to provide draught power, which in turn was a consequence of the changed

political dispensation and government support. Generally it was the wealthier households that still partook in arable agriculture. The reduction in arable agriculture has a big part to play in landscape change. The reduction in cropping and livestock as well as reduced use NTFPs has allowed forest and woodland expansion. Generally, most households viewed the forest and woodland increase as undesirable due to crime risks and the fact that the forest habours more wild animals that raid crops and are seen as dangerous. This study shows that abandoned fields in this region revegetate through time towards a species composition similar to uncleared forests. For the first few decades old fields were dominated by *A. karroo* woodlands which were gradually replaced by forest species, including some invasive alien species. The change in land use and hence forest extent poses interesting challenges for balancing local livelihood needs and conservation priorities in this biodiversity hotspot.

Acknowledgements

Thanks are due to Kathy Cassidy for assistance with the GIS and Nontlantla Busakwe for translation and guidance in the field.

Author Contributions

Conceived and designed the experiments: CMS RTS SES. Performed the experiments: RTS CMS. Analyzed the data: RTS CMS JG. Wrote the manuscript: RTS CMS.

References

1. Bryceson DF, Jamal V (1997) Farewell to farms: De-agrarianisation and Employment in Africa. Aldershot: Ashgate.
2. Diaz GI, Nahuelhual L, Echeverria C, Marin S (2011) Drivers of land abandonment in Southern Chile and implications for landscape planning. Lands Urban Plan 99: 207-217. doi:10.1016/j.landurbplan.2010.11.005.
3. Cramer VA, Hobbs RJ, Standish RJ (2008) What's new about old fields? Land abandonment and ecosystem assembly. Trends Ecol Evol 23(2): 104-112. doi:10.1016/j.tree.2007.10.005. PubMed: 18191278.
4. Weissteiner CJ, Boschetti M, Bottcher K, Carrara P, Bordogna G, Brivio PA (2011) Spatial explicit assessment of rural land abandonment in the Mediterranean area. Glob Planet Change 79: 20-36. doi:10.1016/j.gloplacha.2011.07.009.
5. Khanal NR, Watanabe T (2006) Abandonment of land and its consequences. MT: Res Dev 26 (1): 32-40
6. Schneider LC, Geoghegan J (2006) Land abandonment in an agricultural frontier after a plant invasion: the case of bracken fern in southern Yucatán, Mexico. J Agr Resour Econ 1: 167-177.
7. Norris K, Asase A, Collen B, Gockowksi J, Mason J, Phalan B, Wade A (2010) Biodiversity in a forest-agriculture mosaic: the changing face of West African rainforests. Biol Conserv 143: 2341-2350. doi:10.1016/j.biocon.2009.12.032.
8. Andrew M (1992) A geographical study of agricultural change since the 1930s in Shixini location, Gatyana District, Transkei. Masters in Arts. Grahamstown: Rhodes University. 169 pp.
9. Fay D (2003) The trust is over! We want to plough! Land, livelihoods and reverse resettlement in South Africa's Transkei. Ph.D. of Arts. Boston: Boston University. p. 410 pages
10. De Klerk H (2007) The mutual embodiment of landscape and livelihoods: An environmental history of Nqabara. Masters in Science. Grahamstown: Rhodes University. 192 pp.
11. Andrew M, Fox R (2004) 'Undercultivation' and intensification in the Transkei: a case study of historical changes in the use of arable land in Nompa, Shixini. Dev S Afr 21(1): 687-706.
12. Timmermans HG (2004) Rural livelihoods at Dwesa/Cwebe: Poverty, development and natural resource use on the Wild Coast, South Africa. Maters in Science. Grahamstown: Rhodes University. 188 pp.
13. McAllister PA (2001) Building the homestead: agriculture, labour and beer in South Africa's Transkei. Res S No. 16, Aldershot: African Studies Centre, Ashgate.
14. Hebinck P, Lent PC (2007) Livelihoods and Landscapes: The people of Guquka and Koloni and their resources. Leiden, Brill.
15. Aliber M, Hart TGB (2009) Should subsistence agriculture be supported as a strategy to address rural food insecurity? Agrekon 48(4): 434-458. doi:10.1080/03031853.2009.9523835.
16. Shackleton CM, Shackleton SE, Netshiluvhi TR, Mathabela FR (2005) The contribution and direct-use value of livestock to rural livelihoods in the Sand River catchment, South Africa. Afr J Range For Sci 22: 127-140
17. McCusker B, Carr ER (2006) The co-production of livelihoods and land use change: Case studies from South Africa and Ghana. Geoforum 37: 790-804. doi:10.1016/j.geoforum.2005.09.007.
18. Van Averbeke W, Khosa TB (2007) The contribution of smallholder agriculture to the nutrition of rural households in a semi-arid environment in South Africa. Water S Afr 33: 413-418.
19. Feng D, Hong-Bo S, Lun S, Zong-Sou L, Ming-An S (2007) Secondary succession and its effects on soil moisture and nutrition in abandoned old-fields of hilly region of Loess Plateau, China. Colloids Surf B 58: 278-285. doi:10.1016/j.colsurfb.2007.04.002.
20. Boyes LJ, Gunton RM, Griffiths ME, Lawes MJ (2011) Causes of arrested succession in coastal dune forest. Plant Ecol 212: 21-32. doi:10.1007/s11258-010-9798-6.
21. Nzunda EF (2011) Sprouting, succession and tree species diversity in a South African coastal dune forest. J Trop Ecol 27: 195-203. doi:10.1017/S0266467410000659.
22. Pandolfi JM (2008) Succession. In SE JorgensenB Fath. Encyclopaedia of Ecology. Oxford: Elsevier.
23. Otto R, Krusi BO, Burga CA, Fernandez-Palacios JM (2006) Old-field succession along a precipitation gradient in the semi-arid coastal region of Tenerfe. J Arid Environ 65: 156-178. doi:10.1016/j.jaridenv.2005.07.005.
24. Bonet A (2004) Secondary succession of semi-arid Mediterranean old-fields in south-eastern Spain: insights for conservation and restoration of degraded lands. J Arid Environ 56: 213-233. doi:10.1016/S0140-1963(03)00048-X.
25. von Maltitz GP, van Wyk GF, Everard DA (1999) Successional pathways in disturbed coastal dune forest on the coastal dunes in north-east KwaZulu-Natal, South Africa. S Afr J Bot 62: 188-195.
26. Wassenaar TD, van Aarde RJ, Pimm SL, Ferreira SM (2005) Community convergence in disturbed sub-tropical dune forests. Ecol 86: 655–666. doi:10.1890/03-0836.
27. Berliner D (2011) The conservation status of forests on the Eastern Cape's Wild Coast, South Africa. Fifth Natural Forest and Woodlands Symposia, Richards Bay
28. Lubke RA, Avis AM, Moll JB (1996) Post-mining rehabilitation of costal sand dunes in Zululand, South Africa. Lands Urban Plan 34: 335-345. doi:10.1016/0169-2046(95)00227-8.
29. van Aarde RJ, Ferreira SM, Kritzinger JJ (1996) Successional changes in rehabilitating coastal dune communities in northern KwaZulu-Natal, South Africa. Lands Urban Plan 34: 277-286. doi:10.1016/0169-2046(95)00237-5.
30. Castley GH, Kerly KIH (1996). Paradox Forest Conserv SA For Ecol Mgmt 85: 35-46. doi:10.1016/S0378-1127(96)03748-6.
31. Hayward MW, White RM, Mabandla KM, Bukeye P (2005) Mammalian fauna of indigenous forest in the Transkei region of South Africa, an overdue survey. S Afr J Wildl Res 35: 117-124.
32. Feely JM (1987) The early farmers of Transkei, Southern Africa: before A.D. 1870. BAR International Series, 378. Oxford. Bureau of African Research.
33. Bond WJ, Parr CL (2010) Beyond the forest edge: Ecology, diversity and conservation of the grassy biomes. Biol Conser 143: 2395-2404. doi:10.1016/j.biocon.2009.12.012.
34. Geldenhuys CJ (2004) Concepts and process to control invader plants in and around natural evergreen forest in South Africa. Weed Technol 18: 1386-1391. doi:10.1614/0890-037X(2004)018[1386:CAPTCI]2.0.CO;2.
35. Shackleton CM, Timmermans HG, Nongwe N, Hamer N, Palmer R (2007) Direct-use values of non-timber forest products from two areas on the Transkei Wild Coast. Agrekon 46: 135-156. doi:10.1080/03031853.2007.9523765.

36. Hayward MW (2009) Bushmeat hunting in Dwesa and Cwebe Nature Reserves, Eastern Cape, South Africa. S Afr J Wildl Res 39: 70-84. doi: 10.3957/056.039.0108.
37. Izquierdo AE, Grau R (2009) Agriculture adjustment, land-use transition and protected areas in Northwestern Argentina. J Env Mgmt 90: 858-865. doi:10.1016/j.jenvman.2008.02.013. PubMed: 18439743.
38. von Maltitz GP, Shackleton SE (2004) Use and management of forests and woodlands in South Africa: stakeholders, institutions and processes from past to present. In M LawesH EeleyCM ShackletonB Geach. Indigenous forests and woodlands in South Africa: policy, people and practice. Pietermaritzburg: University of KwaZulu-Natal Press. pp. 109-137.
39. Chalmers N, Fabricius C (2007) Expert and generalist local knowledge about land-cover change on South Africa's Wild Coast: Can local ecological knowledge add value to science? Ecol Soc 12(1): 10.
40. Mangwale K (2010) The impact of forest degradation on carbon stocks of forests in the Matiwane area of the Transkei, South Africa. M.Sc. thesis, Rhodes University, Grahamstown. 120 pp.
41. Bond WJ, Midgley GF (2000) A proposed Co_2-controlled mechanism of woody plant invasion in grasslands and savannas. Glob Change Biol 6: 865-869. doi:10.1046/j.1365-2486.2000.00365.x.
42. Higgins SI, Scheiter S (2012) Atmospheric CO_2 forces abrupt vegetation shifts locally, but not globally. Nature 488 (7410): 209-212. doi:10.1038/nature11238. PubMed: 22763447.
43. Mucina L, Rutherford MC (2006) The vegetation of South Africa. Lesotho and Swaziland. Strelitzia 19 (South African National Biodiversity Institute), Pretoria
44. CEPF (.Critical Ecosystem Partnership Fund) (2010) Ecosystem Profile: Muputaland——Pondoland-Albany Biodiversity Hotspot. Conservation International, Southern African Hotspots Programme and South African National Biodiversity Institute, South Africa
45. Local Municipality Mbashe (2010) Integrated development plan 2010/2011. Dutywa. p. 178.
46. Everatt D, Zulu S (2001) Analysing rural development programmes in South Africa 1994-2001. Dev Update 3: 32-52.
47. Benayas JMR, Martins A, Nicolai JM, Schulz JJ (2007) Abandonment of agricultural land: An overview of drivers and consequences. Persp Agr Vet Sci Nutr Nat Resour 2: 2-14.
48. Bjarnason T, Thorlindsson T (2006) Should I stay or should I go? Migration expectations among youth in Iceland fishing and farming communities. J Rural Stud 22(3): 290-300. doi:10.1016/j.jrurstud.2005.09.004.
49. Wigley BJ, Bond WJ, Hoffman MT (2010) Thicket expansion in a South African savannah under divergent land use: local vs global drivers. Glob Change Biol 16: 964-976. doi:10.1111/j.1365-2486.2009.02030.x.
50. Elands BHM, O'Leary TN, Boerwinkely HWJ, Wiersum KF (2004) Forests as a mirror of rural conditions; local views on the role of forests across Europe. Forest Policy Econ 6: 462-482.
51. Shackleton CM, Shackleton SE (2006) Household wealth status and natural resource use in the Kat River valley, South Africa. Ecol Econ 57(2): 306-317. doi:10.1016/j.ecolecon.2005.04.011.
52. Tognetti PM, Chaneton EJ, Omacini M, Trebino HJ, Leon RJC (2010) Exotic vs. native plant dominance over 20 years of old-field succession on set-aside farmland in Argentina. Biol Conserv 143: 2494-2503. doi: 10.1016/j.biocon.2010.06.016.
53. Prach K, Walker LR (2011) Four opportunities for studies of ecological succession. Trends Ecol Evol 26(3): 119-123. doi:10.1016/j.tree.2010.12.007. PubMed: 21295370.
